# Hepatic-Metabolic Activity of α-Lipoic Acid—Its Influence on Sphingolipid Metabolism and PI3K/Akt/mTOR Pathway in a Rat Model of Metabolic Dysfunction-Associated Steatotic Liver Disease

**DOI:** 10.3390/nu16101501

**Published:** 2024-05-16

**Authors:** Klaudia Sztolsztener, Adrian Chabowski

**Affiliations:** Department of Physiology, Medical University of Bialystok, Mickiewicz 2C Str., 15-222 Bialystok, Poland; adrian.chabowski@umb.edu.pl

**Keywords:** α-lipoic acid, steatosis, ceramide, sphingolipid, liver, insulin, steatohepatitis

## Abstract

Excessive lipid deposition affects hepatic homeostasis and contributes to the development of insulin resistance as a crucial factor for the deterioration of simple steatosis to steatohepatitis. So, it is essential to search for an effective agent for a new therapy for hepatic steatosis development before it progresses to the more advanced stages. Our study aimed to evaluate the potential protective effect of α-lipoic acid (α-LA) administration on the intrahepatic metabolism of sphingolipid and insulin signaling transduction in rats with metabolic dysfunction-associated steatotic liver disease (MASLD). The experiment was conducted on male Wistar rats subjected to a standard diet or a high-fat diet (HFD) and an intragastrically α-LA administration for eight weeks. High-performance liquid chromatography (HPLC) was used to determine sphingolipid content. Immunoblotting was used to measure the expression of selected proteins from sphingolipid and insulin signaling pathways. Multiplex assay kit was used to assess the level of the phosphorylated form of proteins from PI3K/Akt/mTOR transduction. The results revealed that α-LA decreased sphinganine, dihydroceramide, and sphingosine levels and increased ceramide level. We also observed an increased the concentration of phosphorylated forms of sphingosine and sphinganine. Changes in the expression of proteins from sphingolipid metabolism were consistent with changes in sphingolipid pools. Treatment with α-LA activated the PI3K/Akt/mTOR pathway, which enhanced the hepatic phosphorylation of Akt and mTOR. Based on these data, we concluded that α-lipoic acid may alleviate glucose intolerance and may have a protective influence on the sphingolipid metabolism under HFD; thus, this antioxidant appears to protect from MASLD development and steatosis deterioration.

## 1. Introduction

Metabolic dysfunction-associated steatotic liver disease (MASLD) is the most chronic liver disease interpreted by an enhanced lipid accumulation covering at least 5% volume of hepatocytes. As the name suggests, MASLD is known to be closely related to metabolic dysfunction, with the global prevalence of MASLD being about 30% and currently affecting up to 2 billion people in the world [1,2]. It has been recognized that the terminology of fatty liver disease has changed in recent years. Now, the terminology of MASLD includes non-alcoholic fatty liver disease (NAFLD) and alcoholic fatty liver disease (AFLD), in the pathogenesis of which increased alcohol consumption is absent and participates, respectively. It is impossible to distinguish the non-alcoholic and alcoholic causes of fatty liver disorder via histological or ultrasound examinations [3]. In addition to enhanced hepatic lipid storage, the criteria diagnosis and the interpretation of MASLD require the presence of at least one of three additional criteria, namely overweight/obesity, type 2 diabetes mellitus, and also symptoms of metabolic dysregulation [1,3]. It is important to remark that hepatic steatosis results from the disproportion of fatty acid (FA) supply and expenditure that disrupts lipid homeostasis in the liver tissue with the loss of efficiency in metabolizing its overabundance [4,5,6]. Increased dietary fatty acid consumption, exceeding the body’s energy requirements, is primarily stored as triacylglycerol (TAG), which further may be esterified to the more lipotoxic intermediates, the sphingolipid pools such as dihydroceramide (DH-CER) and ceramide (CER) [7,8]. As previous studies have demonstrated, the excessive production and deposition of these molecules deteriorated to simple hepatic steatosis and finally predisposed to cell ballooning and damage [9,10,11].

Moreover, the activation of ceramide synthesis and accumulation in the hepatic parenchyma during the overloaded lipid condition impairs the insulin signaling pathway. Research have considered that ceramide can inhibit protein kinase B (PKB/Akt) and the membrane translocation of glucose transporter (GLUT), suppressing the secretion of insulin and worsening glucose metabolism that favors the development of insulin resistance (IR) and, therefore, liver function deteriorates [9,12]. It is known that an increased hepatic lipid accumulation, obesity, and insulin resistance mediate in the pathogenesis of MASLD and its progression to metabolic dysfunction-associated steatohepatitis (MASH) in which inflammatory processes coexist with fat deposition, and finally can lead to irreversible changes, namely fibrosis, cirrhosis, and hepatocellular carcinoma (HCC) [9,13,14]. Therefore, it is crucial to search for an effective agent for a new therapy for arresting hepatic steatosis development and its progress to the more advanced stages [1]. It seems likely that α-lipoic acid (α-LA), a naturally occurring antioxidant, has a therapeutically effective influence on sphingolipid metabolism and insulin resistance development in the pathogenesis of steatohepatitis. The administration of α-LA as a dietary supplement is expanding in some medical aspects with significant epidemiological and social impact, like diabetes mellitus and hypertension [15]. α-LA is also considered as substance that enhances glucose uptake by GLUT translocation into the plasmatic membrane (an effect observed in adipocytes and skeletal muscle cells) [15,16,17]. Our previous data suggest that the administration of α-LA attenuates insulin resistance, which is reflected in reduced levels of plasma glucose and insulin. The same research points to reductions in the rats’ body weight after this antioxidant treatment. The diminishment in the two mentioned significant factors of MASLD diagnosis suggests the protective influence of α-lipoic acid on liver disorders related to steatosis occurrence [18]. Notably, our other study revealed the effect of α-LA supplementation on limiting the development of hepatic inflammation under lipid overload condition, suggesting a positive correlation between the treatment with this antioxidant and the improvement in the liver structure and function [19]. In this study, we will try to critically analyze a new perspective of MASLD treatment by examining changes in the sphingolipid metabolism and insulin pathway in which they are involved, to deepen knowledge about the influence of α-LA on hepatic tissue functioning. We will determine the effect of α-LA on the level of selected sphingolipid fractions and the expression of proteins that regulate their metabolism. In this study, we will also examine how α-LA administration affects the intrahepatic content of proteins from the insulin signaling pathway. We will identify the “breaking point” in the IR condition, which predisposes to steatosis deterioration and requires the stratification of MASLD treatment.

## 2. Materials and Methods

### 2.1. Experimental Model and Procedures

This experiment was conducted on male Wistar rats with an initial body weight of approximately 50–70 g. Animals were kept with unrestricted access to standard rodent chow and water under holding conditions (a reverse 12 h light/dark cycle, at 22 ± 2 °C air temperature and with 55 ± 10% humidity). After one week of acclimatization rats were randomly divided into four groups (ten rats per each experimental group) as follows: (1) control—rats received a standard rodent diet with a regular content of fat (kcal distribution: 65.5% carbohydrate, 24.2% protein and 10.3% fat; Agropol, Motycz, Poland); (2) HFD—rats received a high-fat diet (HFD; kcal distribution: 59.8% fat, 20.1% protein and 20.1% carbohydrate; Research Diet, New Brunswick, NJ, USA); (3) α-LA—rats received a standard rodent diet with the supplementation of α-lipoic (α-LA; Sigma Aldrich, Saint Louis, MO, USA); (4) HFD + α-LA—rats received a high-fat diet with α-lipoic supplementation. The concentration of α-LA was estimated at 30 mg/kg of body weight measured every two days during the eight week experiment. The antioxidant solution was prepared by dissolving α-LA powder in saline and then intragastrically administering it once daily. The intragastric infusion of α-LA solution was conducted using an oral gavage feeding tube for rodents inserted through the mouth directly into the stomach. During the experiment, none of the animals were hurt or died. At the end of the eight weeks of the experiment, rats were anesthetized with a phenobarbital injection (intraperitoneal administration at a dose of 80 mg/kg of body weight). During the procedure for euthanasia, the animals were gently restrained and an appropriate size of needle was used; then, the calculated anesthetic dose of sodium pentobarbital was injected. Following the injection, animals were euthanized by bleeding them to death (from the inferior vena cava). The liver tissue was immediately removed and frozen at a liquid nitrogen temperature using precooled aluminum tongs, then stored at −80 °C until appropriate determinations.

The experimental study was performed after obtaining the approval of the Local Ethical Committee for Animal Experiments at the Medical University of Bialystok (approval No. 21/2017).

### 2.2. The Determination of Hepatic Sphingolipid Content

The hepatic content of sphinganine (SFA), sphinganine-1-phosphate (SA1P), dihydroceramide (DH-CER), ceramide (CER), sphingosine (SFO) and sphingosine-1-phosphate (S1P) were determined using the high-performance liquid chromatography (HPLC) method. Firstly, the liver tissue was homogenized, and then in the presence of internal standard containing C17-sphingosine-1-phosphate and C17-sphingosine (Avanti Polar Lipids, Alabaster, AL, USA) lipids from the obtained homogenates were ultrasonicated and extracted with the addition of chloroform solution. After the dephosphorylation to sphinganine and sphingosine, the content of sphingoid base-1-phosphates was assessed by the use of alkaline phosphate (Sigma Aldrich, Saint Louis, MO, USA). Following the transfer of small aliquots into new tubes containing an internal standard like N-palmitoyl-D-erythro-sphingosine (C17-base), the samples were subjected to an alkaline hydrolysis procedure to convert CER to SFO. The hepatic content of dephosphorylated sphingoid bases, free sphinganine, free sphingosine and also sphingosine sourced from ceramide were changed into their o-phthalaldehyde derivatives and measured with the use of HPLC system (Varian ProStar; Agilent Technologies, Santa Clara, CA, USA) with a fluorescence detector and C18 reversed-phase column (OmniSpher 5; Varian Inc., Palo Alto, CA, USA; 4.6 × 150 mm).

Briefly, the HPLC method was validated for obtaining repeatable and reliable results based on the International Council for Harmonization (ICH) guidelines. Sensitivity is defined as the limit of detection (LOD) and limit of quantitation (LOQ). The assay of the amount of peak height with signal-to-noise (S/N) of three (for LOD) and the amount of a peak with S/N > 10 (for LOQ). Running the HPLC procedure blank and placebo extract was used as the reference material (a “cocktail” or retention marker solution) to ensure exact results that assessed an analyte accurately in a sample; synthetic precursors, enantiomers and excipients were used to provide the technique’s selectivity. For a valid method, standard deviation (SD) or relative standard deviation (RSD) estimated the assaying of a sufficient number of homogenous sample aliquots to be determined. Precision was determined by multiple (at least five replicates) injections of the same reference solution and the acceptable value of peak area precision was a mandatory requirement for the quantitative HPLC analysis. Moreover, the accuracy of the HPLC technique was evaluated by determining the recovery of the spiked analytes to the sample matrix, namely diluent for testing drug substances (DS) and placebo for testing of drug product (DP) [20].

### 2.3. The Determination of Hepatic Expression of Selected Proteins Involved in Sphingolipid and Insulin Signaling Pathways

The expression of proteins regulating the sphingolipid metabolism pathway, i.e., serine palmitoyltransferase 1 (SPTLC1; Santa Cruz Biotechnology, Inc., Dallas, TX, USA), serine palmitoyltransferase 2 (SPTLC2; Santa Cruz Biotechnology, Inc., Dallas, TX, USA), ceramide synthase 2 (CerS2; Abcam, Cambridge, UK), ceramide synthase 4 (CerS4; Abcam, Cambridge, UK), ceramide synthase 6 (CerS6; Abcam, Cambridge, UK), sphingosine kinase 1 (SPHK1; Sigma Aldrich, Saint Louis, MO, USA), sphingosine kinase 2 (SPHK2; Sigma Aldrich, Saint Louis, MO, USA), acid ceramidase (ASAH1; Santa Cruz Biotechnology, Inc., Dallas, TX, USA), neutral ceramidase (ASAH2; Santa Cruz Biotechnology, Inc., Dallas, TX, USA), alkaline ceramidase (ASAH3; Santa Cruz Biotechnology, Inc., Dallas, TX, USA), alkaline sphingomyelinase (Alk-SMase; Santa Cruz Biotechnology, Inc., Dallas, TX, USA), neutral sphingomyelinase (N-SMase; Santa Cruz Biotechnology, Inc., Dallas, TX, USA) and protein involved in insulin signaling transduction, i.e., glucose transporter 2 (GLUT2; Santa Cruz Biotechnology, Inc., Dallas, TX, USA) was determined by the Western blot technique.

Before appropriate determination, the liver tissue was homogenized in radioimmunoprecipitation assay (RIPA) buffer with the addition of protease and phosphatase inhibitors (Roche Diagnostic GmbH, Mannheim, Germany). The supernatants were transferred into fresh tubes after centrifugation at 10,000× *g* at 4 °C for 30 min. In this fraction, the total protein concentration was assessed using a bicinchoninic acid (BCA) method with bovine serum albumin (BSA) as a standard. After that, homogenates were restored using a Laemmli buffer (Bio-Rad, Hercules, CA, USA) for the same protein mass in each sample. The prepared samples were loaded on the Criterion TGX Stain-Free Precast Gels (Bio-Rad, Hercules, CA, USA), separated during electrophoresis procedure, and also transferred on the nitrocellulose membrane for the wet transfer system or transferred on the polyvinylidene fluoride (PVDF) membrane for the semi-dry transfer system. After the incubation with the blocking buffer (5% BSA or 5% non-fat dry milk prepared in Tris-buffered saline with the addition of Tween-20 (TBST)), membranes were immunoblotted overnight with the appropriate above-mentioned primary antibodies. On the second day, the secondary antibodies conjugated with horseradish peroxidase (HRP) were used to immunoblot membranes. Finally, the protein bands were visualized using a chemiluminescent Clarity Western ECL Substrate (Bio-Rad, Hercules, CA, USA). Then, the obtained signals were measured densitometrically using the ChemiDoc visualization system (Image Laboratory 6.0.1 Software; Bio-Rad, Warsaw, Poland). The protein expression was standardized to the total protein expression as a control, which was set as 100%.

### 2.4. The Determination of Hepatic Content of Phosphoproteins Involved in Insulin Transduction Pathway

The hepatic level of phosphorylated proteins from the insulin signaling pathway, i.e., insulin receptor substrate 1 (pIRS1(Ser636/Ser639)), phosphate and tensin homolog (pPTEN(Ser380)), protein kinase B (pAkt(Ser473)), glycogen synthase kinase 3α/β (pGSK3α/β(Ser21/Ser9)), Bcl-2-associated agonist of cell death (pBAD(Ser136)), mechanistic target of rapamycin (pmTOR(Ser2448)), P70 ribosomal protein S6 kinase (pP70 S6 kinase(Thr389)) and S6 ribosomal protein (pS6RP(Ser235/Ser236)) was assessed using a Bio-Plex Pro Cell Signaling Assays (Bio-Rad, Hercules, CA, USA).

Before appropriate determination, the lysates of liver tissue were prepared in cell lysis buffer (Bio-Rad, Hercules, CA, USA) with the addition of cell lysis factor QG (Bio-Rad, Hercules, CA, USA) and phenylmethylsulfonyl fluoride (PMSF; Sigma Aldrich, Saint Louis, MO, USA) and then were centrifuged at 15,000 × *g* at 4 °C for 10 min. After the transfer of the obtained supernatant into a new tube, the protein concentration was measured and ultimately set in the range of 20–200 µg/mL. The supernatant samples were stored at −80 °C for further measurement.

The immunoassay multiplex flow procedure was based on the determination of using covalent magnetic beads to bind the detection antibody. In brief, diluted coupled beads were pipetted to all wells, and then the 96-well plate was washed two times. Blanks, cell lysis controls and all samples were added into the appropriate well, and the plate was incubated overnight with shaking in the dark. On the next day, following washing three times, the detection antibody was pipetted and the plate was incubated for 30 min with shaking in the dark and then washed three times. The streptavidin-phycoerythrin (SA-PE) conjugate was added to each well, and the plate was incubated for 10 min, shaking in the dark. Finally, following a series of washes and the addition of resuspension buffer for beads and short shaking (30 s), the concentration of intrahepatic phosphorylated proteins was measured using a Bio-Plex 200 System equipped with Bio-Plex Manager 6.0 Software (Bio-Rad, Hercules, CA, USA).

### 2.5. Data Analyzes

The statistical analyses of data obtained in our experiment were conducted using GraphPad Prism 8.2.1 Software (San Diego, CA, USA). The results are presented as mean ± standard deviation (SD) and based on ten independent determinations, except data from immunoblotting analysis (based on six independent decisions). The variance homogeneity and value distribution were assessed using Shapiro–Wilk and Bartlett’s tests. The statistical comparison between experimental groups was estimated using two-way ANOVA encompassing Tukey’s test and parametric *t*-test or non-parametric Mann–Whitney U test appropriately for differences with normal and abnormal distribution. Statistically significant differences were considered as a *p*-value < 0.05.

## 3. Results

### 3.1. α-Lipoic Influence on the Sphingolipid Content in the Liver Tissue of Rats Subjected to a Standard Diet or a High-Fat Diet

**Figure 1:**
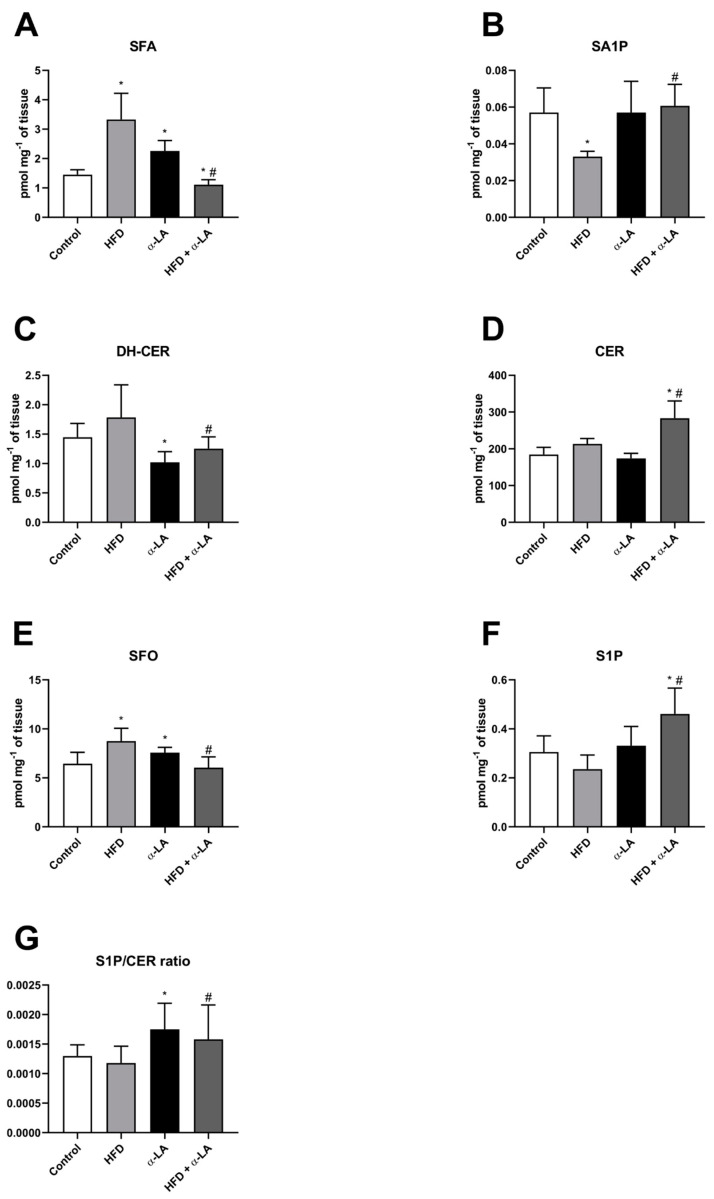
The hepatic content of selected sphingolipid fractions, i.e., sphinganine (SFA, (**A**)), sphinganine-1-phosphate (SA1P, (**B**)), dihydroceramide (DH-CER, (**C**)), ceramide (CER, (**D**)), sphingosine (SFO, (**E**)), sphingosine-1-phosphate (S1P, (**F**)) and sphingosine-1-phosphate/ceramide ratio (S1P/CER ratio, (**G**)) in rats subjected to a standard diet (Control) or a high-fat diet (HFD) after α-lipoic acid (α-LA) administration for eight weeks. The concentration of sphingolipids was measured using high-performance liquid chromatography (HPLC). The results are presented as mean ± standard deviation (SD) and established on ten independent determinations in each experimental group. * *p* < 0.05—significant differences vs. Control group. ^#^ *p* < 0.05—significant differences vs. HFD group.

In the liver tissue homogenates, we observed an increase in the sphingosine (SFA) concentration in the high-fat diet (HFD) and α-lipoic acid (α-LA) groups (HFD: +128.9%, α-LA: +55.6%; vs. Control group; *p* < 0.05; Figure 1A). α-lipoic acid treatment reduced the hepatic level of SFA in rats subjected to an HFD (HFD + α-LA: −23.6% and −66.6%; vs. Control and HFD groups, respectively; *p* < 0.05; Figure 1A). Moreover, high-fat feeding decreased the sphinganine-1-phosphate (SA1P) content (HFD: −42.1%; vs. Control group; *p* < 0.05; Figure 1B), which was increased after α-lipoic acid supplementation (HFD + α-LA: +84.0%; vs. HFD group; *p* < 0.05; Figure 1B). We also noticed a significant decline in the content of dihydroceramide (DH-CER) as a result of rats treatment with α-LA alone and in combination with an HFD (α-LA: −29.6%, HFD + α-LA: −29.8%; *p* < 0.05; Figure 1C) in comparison with the Control and HFD groups, respectively. In the case of ceramide (CER) level, an expansion in this parameter was provoked by an HFD with α-LA administration (HFD + α-LA: +53.5% and +32.7%; vs. Control and HFD groups, respectively; *p* < 0.05; Figure 1D). In our study, sphingosine (SFO) content was also raised in two experimental groups (HFD: +36.0%, α-LA: +17.5%; *p* < 0.05; Figure 1E) concerning the Control group. Rats subject to an HFD and α-LA had a decline in the hepatic content of SFO (HFD + α-LA: −31.0%; vs. HFD group; *p* < 0.05; Figure 1E). In the same experimental group, the sphingosine-1-phosphate (S1P) concentration was enhanced (HFD + α-LA: +50.7% and +95.3%; *p* < 0.05; Figure 1F) more than in the Control and HFD groups, respectively. Additionally, the ratio of S1P/CER was enhanced in both groups treated with α-lipoic acid (α-LA: +45.2%, HFD + α-LA: +74.2%; vs. Control and HFD groups, respectively; *p* < 0.05; Figure 1G).

### 3.2. α-Lipoic Influence on the Expression of Proteins from Sphingolipid Pathway in the Liver Tissue of Rats Subjected to a Standard Diet or a High-Fat Diet

**Figure 2:**
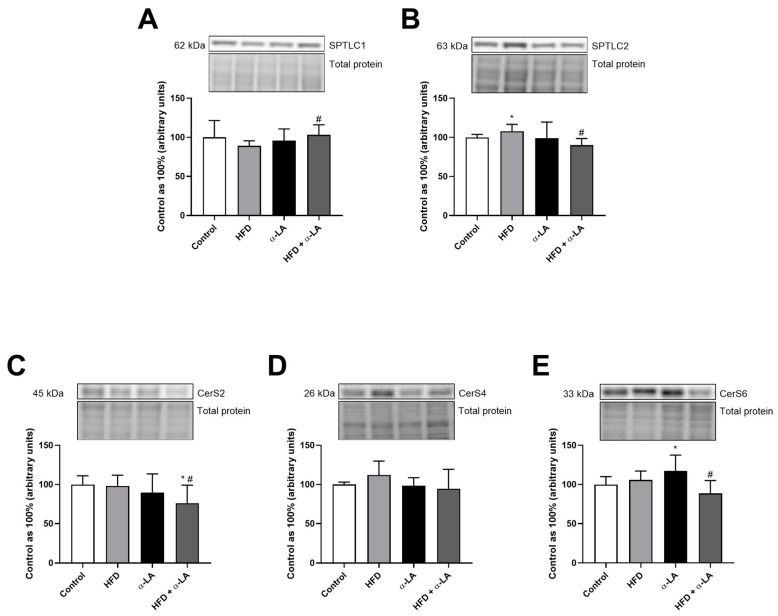
The hepatic expression of proteins from the ceramide *de novo* synthesis pathway, i.e., serine palmitoyltransferase 1 (SPTLC1, (**A**)), serine palmitoyltransferase 2 (SPTLC2, (**B**)) and enzymes that share salvage and *de novo* synthesis pathways, i.e., ceramide synthase 2 (CerS2, (**C**)), ceramide synthase 4 (CerS4, (**D**)) and ceramide synthase 6 (CerS6, (**E**)) in rats subjected to a standard diet (Control) or a high-fat diet (HFD) after α-lipoic acid (α-LA) administration for eight weeks. The Western blot measured the expression of proteins from the sphingolipid pathway. The results are presented as mean ± standard deviation (SD) and established on six independent determinations in each experimental group. * *p* < 0.05—significant differences vs. Control group. ^#^ *p* < 0.05—significant differences vs. HFD group.

In the liver tissue homogenates, we observed an increase in the expression of serine palmitoyltransferase 1 (SPTLC1) after treatment with HFD and α-LA (HFD + α-LA: +15.7%; vs. HFD group; *p* < 0.05; Figure 2A). Moreover, the expression of serine palmitoyltransferase 2 (SPTLC2) was raised under the lipid overload condition (HFD: +7.9%; vs. control group; *p* < 0.05; Figure 2B), but this change was abolished by administration of α-LA (HFD + α-LA: −16.5%; vs. HFD group; *p* < 0.05; Figure 2B). In the HFD + α-LA group, we noticed a reduction in the ceramide synthase 2 (CerS2) expression (HFD + α-LA: −23.8% and −22.4%; *p* < 0.05; Figure 2C) in comparison with the Control and HFD groups, respectively. Ceramide synthase 4 (CerS4) expression was unchanged in all experimental groups (Figure 2D). The expression of ceramide synthase 6 (CerS6) was higher in rats from the α-LA group (α-LA: +17.2%; vs. Control group; *p* < 0.05; Figure 2E) and was lower in rats from the HFD + α-LA group (HFD + α-LA: −16.2%; vs. HFD group; *p* < 0.05; Figure 2E).

**Figure 3:**
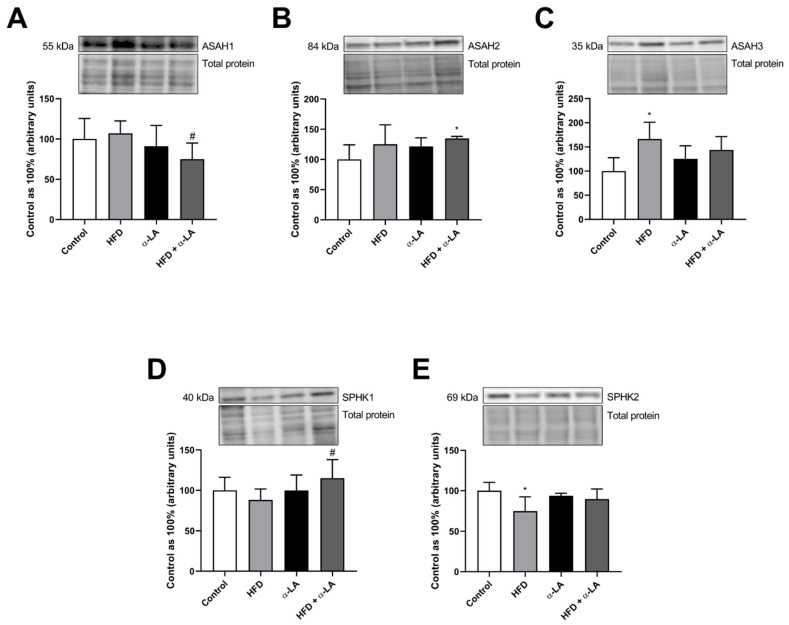
The hepatic expression of proteins from the sphingolipid catabolism pathway, i.e., acid ceramidase (ASAH1, (**A**)), neutral ceramidase (ASAH2, (**B**)), alkaline ceramidase (ASAH3, (**C**)), sphingosine kinase 1 (SPHK1, (**D**)) and sphingosine kinase 2 (SPHK2, (**E**)) in rats subjected to a standard diet (Control) or a high-fat diet (HFD) after α-lipoic acid (α-LA) administration for eight weeks. The Western blot measured the expression of proteins from the sphingolipid pathway. The results are presented as mean ± standard deviation (SD) and established on six independent determinations in each experimental group. * *p* < 0.05—significant differences vs. Control group. ^#^ *p* < 0.05—significant differences vs. HFD group.

The expression of acid ceramidase (ASAH1) was lower in obese rats after α-lipoic acid administration (HFD + α-LA: −29.8%; *p* < 0.05; Figure 3A) than in the rats from the HFD group. In the HFD + α-LA group, we observed an enhancement in the neutral ceramidase (ASAH2) expression (HFD + α-LA: +34.7%; *p* < 0.05; Figure 3B) concerning the rats subjected to a standard rodent chow. Furthermore, the expression of alkaline ceramidase (ASAH3) was higher in rats under an HFD (HFD: +66.4%; *p* < 0.05; Figure 3C) than in rats from the Control group. Treatment with α-lipoic acid enhanced the expression of sphingosine kinase 1 (SPHK1) in animals subjected to an HFD (HFD + α-LA: +30.7%; *p* < 0.05; Figure 3D). Additionally, we noticed a decrease in the expression of sphingosine kinase 2 (SPHK2) in the HFD group (HFD: −25.3%; *p* < 0.05; Figure 3E) compared to the Control group.

**Figure 4:**
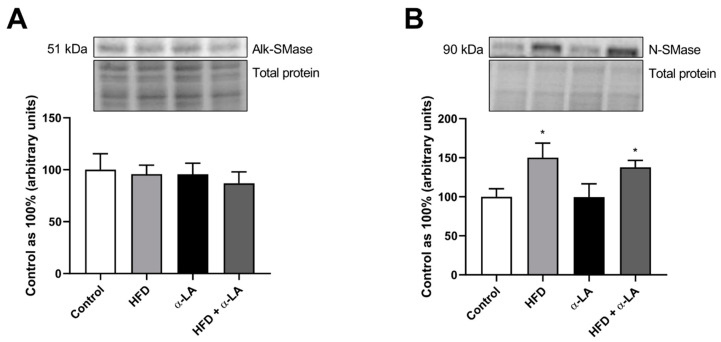
The hepatic expression of proteins from the sphingomyelin pathway, i.e., alkaline sphingomyelinase (Alk-SMase, (**A**)) and neutral sphingomyelinase (N-SMase, (**B**)) in rats subjected to a standard diet (Control) or a high-fat diet (HFD) after α-lipoic acid (α-LA) administration for eight weeks. The Western blot measured the expression of proteins from the sphingolipid pathway. The results are presented as mean ± standard deviation (SD) and established on six independent determinations in each experimental group. * *p* < 0.05—significant differences vs. Control group.

The expression of alkaline sphingomyelinase (Alk-SMase) was unchanged in all experimental groups. In the lipid overload condition, the expression of neutral sphingomyelinase (N-SMase) was enhanced (HFD: +50.0%, HFD + α-LA: +37.8%; *p* < 0.05; Figure 4B) concerning rats from the Control group.

### 3.3. α-Lipoic Influence on the Phosphorylation State of Proteins from Insulin Signaling Pathway in the Liver Tissue of Rats Subjected to a Standard Diet or a High-Fat Diet

**Figure 5:**
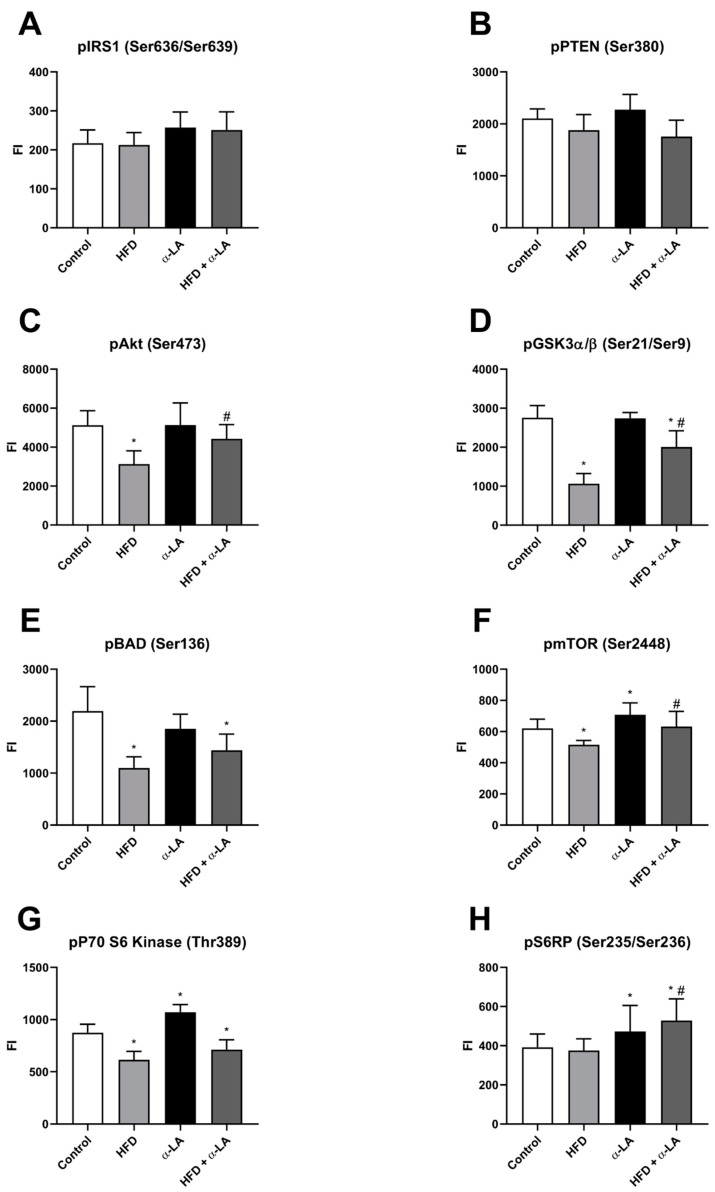
The hepatic content of phosphorylated proteins from insulin transduction pathway, i.e., insulin receptor substrate 1 (pIRS1(Ser636/Ser639), (**A**)), phosphate and tensin homolog (pPTEN(Ser380), (**B**)), protein kinase B (pAkt(Ser473), (**C**)), glycogen synthase kinase 3α/β (pGSK3α/β(Ser21/Ser9), (**D**)), Bcl-2-associated agonist of cell death (pBAD(Ser136), (**E**)), mechanistic target of rapamycin (pmTOR(Ser2448), (**F**)), P70 ribosomal protein S6 kinase (pP70 S6 kinase(Thr389), (**G**)) and S6 ribosomal protein (pS6RP(Ser235/Ser236), (**H**)) in rats subjected to a standard diet (Control) or a high-fat diet (HFD) after α-lipoic acid (α-LA) administration for eight weeks. Magnetic bead-related immunoassay-measured phosphorylated proteins’ fluorescent intensity (FI). The results are presented as mean ± standard deviation (SD) and established on ten independent determinations in each experimental group. * *p* < 0.05—significant differences vs. Control group. ^#^ *p* < 0.05—significant differences vs. HFD group.

The phosphorylated form of insulin receptor substrate 1 (pIRS1(Ser636/Ser639)) and phosphate and tensin homolog (pPTEN(Ser380)) were unchanged in all experimental groups (Figure 5A and Figure 5B, respectively). The hepatic content of phosphorylated protein kinase B (pAkt(Ser473)) was attenuated in rats who received an HFD alone (HFD: −38.9%; vs. Control group; *p* < 0.05; Figure 5C). Importantly, α-lipoic acid supplementation provoked a significant increase in the pAkt level in the lipid overload condition (HFD + α-LA: +41.5%; vs. HFD group; *p* < 0.05; Figure 5C). A high-fat diet feeding also provoked an impairment in fluorescent intensity of the phosphorylated glycogen synthase kinase 3α/β (pGSK3α/β(Ser21/Ser9)) (HFD: −61.3%, HFD + α-LA: −27.2%; *p* < 0.05; Figure 5D) in relation to the Control rats. The rats treated with an HFD in the combination with α-LA had a higher level of pGSK3α/β (HFD + α-LA: +88.0%; *p* < 0.05; Figure 5D) than in the HFD group. In our study, we also noticed an diminishing in the content of phosphorylated Bcl-2-associated agonist of cell death (pBAD(Ser136)) in rats fed an HFD (HFD: −49.9%, HFD + α-LA: −34.4%; vs. Control group; *p* < 0.05; Figure 5E). In all experimental groups, significant changes were observed in the phosphorylated mechanistic target of rapamycin (pmTOR(Ser2448)) level (HFD: −16.9%, α-LA: +14.1%, HFD + α-LA: +22.6%; vs. Control or HFD groups; *p* < 0.05; Figure 5F) and in the phosphorylated P70 ribosomal protein S6 kinase (pP70 S6 kinase(Thr389)) level (HFD: −29.7%, α-LA: +22.4%, HFD + α-LA: −18.6%; vs. Control group; *p* < 0.05; Figure 5G). Additionally, the supplementation of antioxidants to rats that received a standard diet and also a high-fat diet caused an increment in the phosphorylated S6 ribosomal protein’s (pS6RP(Ser235/Ser236)) fluorescent intensity (α-LA: +20.8%, HFD + α-LA: +34.9% and +40.7%; vs. Control and HFD groups; *p* < 0.05; Figure 5H).

### 3.4. α-Lipoic Influence on the Expression of Protein from Insulin Signaling Pathway in the Liver Tissue of Rats Subjected to a Standard Diet or a High-Fat Diet

**Figure 6:**
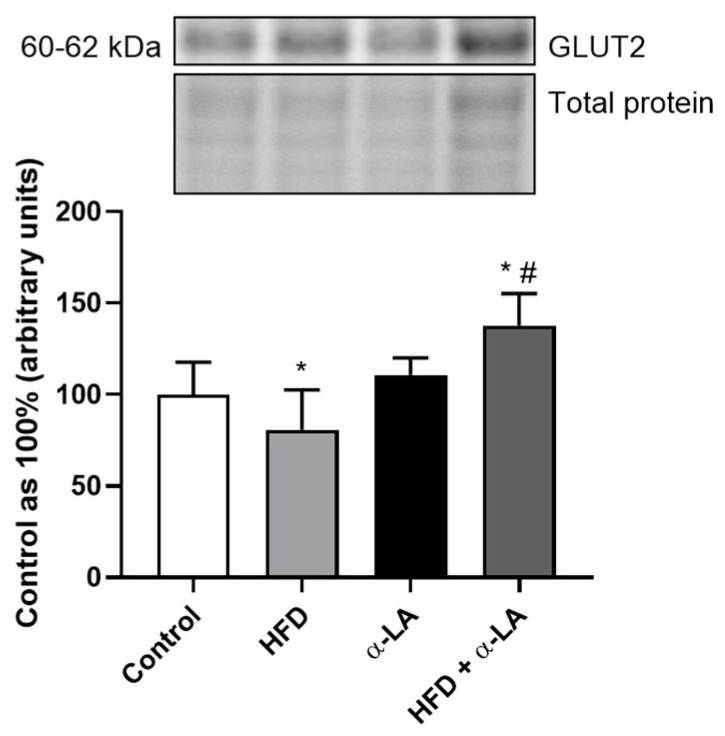
The hepatic expression of protein from insulin signaling pathway, i.e., glucose transporter 2 (GLUT2) in rats subjected to a standard diet (Control) or a high-fat diet (HFD) after α-lipoic acid (α-LA) administration for eight weeks. The Western blot measured the expression of protein from the insulin signaling pathway. The results are presented as mean ± standard deviation (SD) and established on six independent determinations in each experimental group. * *p* < 0.05—significant differences vs. Control group. ^#^ *p* < 0.05—significant differences vs. HFD group.

In the liver tissue homogenates, the expression of glucose transporter 2 (GLUT2) decreased after high-fat diet feeding (HFD: −19.3%; vs. Control group; *p* < 0.05; Figure 6) and then rose after α-LA supplementation (HFD + α-LA: +37.8% and +70.7%; vs. Control and HFD groups, respectively; *p* < 0.05; Figure 6).

## 4. Discussion

This study elucidates how α-LA influences on the sphingolipid and insulin transduction pathways in the development of metabolic-associated steatotic liver disease induced by high-fat feeding.

Increasing evidence conducted on animal models indicated that an excessive delivery of FAs is associated with impairment in lipid metabolism at multiple levels. As was demonstrated by Parker et al., in the lipid overload condition, the excess free fatty acid (FFA) pool is shunted to the sphingolipid *de novo* synthesis pathway. For this pathway, ceramide is a key intermediate that has lipotoxicity properties. An intensification in the ceramide formation is strongly destructive for liver cells and may inhibit insulin sensitivity [12]. Our study did not observe a significant change in the ceramide level after a high-calorie diet administration. A lack of significant increase in the ceramide level after high-fat feeding may result from the different cell adaptations to buffer the increased FA levels in the circulation and storage in the hepatocytes’ cytoplasm spaces [5,6]. Following this non-significant alteration in ceramide content, a study conducted by Kim et al. revealed that the hepatic C16 ceramide level was elevated only at 24 weeks of an HFD [21]. However, despite a statistically significant ceramide level, we noticed an increase in the expression of the first hepatic-specific protein rate-limiting *de novo* synthesis pathway, like serine palmitoyltransferase 2 (SPTLC2) following an HFD. This observation was supported by an enhanced sphinganine (SFA) level, which may then be metabolized into dihydroceramide or phosphorylated into sphinganine-1-phosphate (SA1P). The first process is regulated by the expression of proteins sharing *de novo* and salvage pathways, namely ceramide synthases (CerS), each isoform preferring different fatty acyl chain lengths, but the second pathway is associated with the family of enzymes, namely sphingosine kinase (SPHK) [22]. In this study, feeding with a high-fat diet also caused slight (non-significant) intensification of dihydroceramide formation, so we can suppose that the *de novo* ceramide pathway was exacerbated only to the SFA synthesis (two times increase) in obese rats. However, the present study, for the first time, presented the role of α-lipoic acid in the regulation of sphingolipid metabolism in diet-induced simple hepatic steatosis. The treatment with α-lipoic acid decreased the expression of hepatic-specific SPTLC2, which was reflected in the rate-limiting *de novo* synthesis pathway, like depressed sphinganine and dihydroceramide concentrations in the liver tissue of rats subjected to a high-fat diet. Our study also revealed that the supplementation of antioxidants decreased the dihydroceramide content through the depletion of CerS expression, simultaneously enhancing ceramide content. These changes may result from increased dihydroceramide desaturase (DES) expression and may be indirectly associated with activating salvage or sphingomyelin pathways [23]. We can suppose that α-lipoic acid is shown to improve (but not completely normalize) the sphingolipid-related liver functioning in diet-induced steatosis. Furthermore, the mentioned effects in CerS expression, especially decreased in the ceramide synthase 6 (CerS6) expression after α-LA supplementation, are most consistent with the available data indicating that CerS6 deficiency in mice model decreased the C16 ceramide content, protecting from glucose intolerance and obesity induced by an HFD [24]. CerS6-deficient animals also have reduced macrophage infiltration and mRNA expression of pro-inflammatory genes, suggesting its role in inhibiting inflammation development and promoting immune functioning [23,24]. As previously presented, salvage (recycling) and sphingomyelinase pathways produce at least 50% to even 90% of sphingolipid mediators [22]. First of all, under an HFD, the sphingosine (SFO) level was increased and supported by overexpression of alkaline ceramidase (ASAH3) and the trend was toward an increase in hepatic-specific neutral ceramidase (ASAH2) expression; thus, these changes could also explain the lack of significant change in ceramide content as a result of increased SFO synthesis from CER under ceramidase activation. We determined that α-LA was unchanged at the significant level of the expression of proteins from the recycling metabolic pathway. This assumption was supported by a decreased SFA level and an increased CER level shown in the data below. It should be noted that an impairment in the sphingosine bases (sphingosine and sphinganine) content observed in rats treated with a high-fat diet and antioxidant may be associated with their phosphorylation into sphinganine-1-phosphate and sphingosine-1-phosphate (S1P), respectively [22,25]. Ishay et al. reported that sphingolipids, especially CER and S1P, play an important role in liver tissue’s chronic inflammation and fibrosis development. Importantly, S1P-regulated parenchymatic and immune cells are driven into spaces of cells with active inflammation, which delays the pathogenesis of hepatitis; therefore, S1P may constitute the potential target for the treatment of simple steatosis and its deterioration to active steatotic-related inflammation [26]. In this study, we observed an elevation in the phosphorylated form of SFA and SFO content after α-lipoic acid supplementation in obese rats. Our observation is in line with the results from a study conducted by Prieto-Hontoria et al., which showed that lipoic acid treatment inhibited glucose-induced leptin secretion from adipocytes by the significantly activated SFO phosphorylation to S1P, which resulted in the activation of phosphoinositide 3-kinase (PI3K) insulin transduction and glucose metabolism [27]. Moreover, the phosphorylation of sphingosine bases is a pivotal process in cell biology, promoting survival, proliferation and migration [23]. To confirm the protective role and consequences of α-lipoic acid treatment, increased S1P and SA1P levels have limited inflammation and immune responses during the progression of MASLD to MASH [23,28]. Additionally, in regulating lipid metabolism during hepatic injury, the balance between sphingosine-1-phosphate and ceramide also plays an important role. These sphingolipid species have opposite effects; S1P enhances cells’ survival, proliferation and metabolism, but CER amplifies the inflammatory responses and cell growth arrest and apoptosis [9,29,30,31]. The protective role of antioxidant treatment in this study was confirmed by an enhancement in the hepatic value of the S1P/CER ratio. It may suggest that α-lipoic acid reduced hepatic injury occurrence and the imbalance of bioactive sphingolipid species.

In response to the extensive availability of fatty acids, the hepatic content of phosphorylated Akt at residue Ser473 was decreased, which is supposed to aggravate insulin signaling in the liver tissue of rats with MASLD. Data suggest that enhanced C16 ceramide deposition may cause the deterioration in Akt phosphorylation and weaken hepatocytes’ insulin sensitivity [32,33]. As mentioned in this study, we did not observe a significant ceramide concentration under high-fat feeding (numerous studies indicated increased CER content only after 10, 12 and 24 weeks of HFD) [23,34,35,36]. This discrepancy may be associated with the decreased pAkt level being noticed in the first week of high-caloric feeding without changes in the hepatic ceramide content (data published in our previous study) [37]. We also observed that in rats fed a high-fat diet, the hepatic expression was reduced, which may be associated with the increased glucose circulation concentration, thus worsening insulin sensitivity and favoring steatosis deterioration. Data describing plasma insulin and glucose levels with HOMA-IR in this model were published in our previously study [18]. Prominently, the supplementation of α-lipoic acid increased the phosphorylation of Akt. Our observation is consistent with that of Tardif et al., and the results of Gomes et al. presented that α-LA increased the activity of proteins from insulin signaling pathways like pAkt and the translocation of glucose transporters into cell membranes of adipocytes and muscle cells [15,38,39]. We may suggest that this antioxidant’s properties include activating insulin transduction by inducing hepatocytes’ glucose uptake and enhancing semi-mediators of insulin signaling (pAkt) [40], which was reflected in the reduction in plasma glucose and insulin levels (data published in our study) [18]. The decline in the phosphorylation of Akt after HFD administration depressed the level of phosphorylated mechanistic target of rapamycin (pmTOR) at residue Ser2448. Importantly, mTOR regulation is provided by the liver, an essential metabolic organ that maintains structural and functional homeostasis. The failure in the level of pmTOR may dysregulate lipogenesis and accelerate the anabolism of macromolecules, such as lipids and proteins [41,42]. A novel finding in this study is that treatment with α-lipoic acid in the Akt-dependent phosphorylation site on mTOR is a strong steatohepatitis protector, leading to enhanced glucose and insulin tolerance and finally hypoglycemia [41,42,43], which was also noticed in our previously published data [18]. Evidence also demonstrated that α-lipoic acid limited the formation of autophagosome, in which the downstream target S6 ribosomal protein (pS6RP(Ser235/Ser236)) also mediates [43]. Indeed, α-LA-treated obese rats had an elevated level of phosphorylated S6RP, which may reveal upregulated protein translation and result in hepatocyte proliferation. We also suspect that the increment in pS6RP may reflect an inhibitory effect on the development of apoptotic effect in the liver tissue under HFD feeding [44]. In the HFD + α-LA group, despite the lack of changes in the level of phosphorylated P70 ribosomal protein S6 kinase (pP70 S6 kinase(Thr389)), we noticed increases in the phosphorylation of S6RP. We suspect that an enhanced pS6RP level may result from an increment in another isoform of S6 kinase, like P90, after antioxidant exposition that regulates the phosphorylation of ribosomal protein [44]. A pivotal role in disrupting insulin signaling in the liver tissue also maintains the level of phosphorylated glycogen synthase kinase 3α/β (pGSK3α/β(Ser21/Ser9)). In our study, the antioxidant treatment caused an increase in the pGSK3α/β content in rats with obesity provoked by HFD feeding. It is worth noting that the mentioned alteration may be a consequence of the enhanced pAkt level and may provoke the promotion of nuclear factor erythroid 2-related factor 2 (Nrf-2) response, protecting the inflammation development from HFD-induced steatosis and its progression to hepatitis [45], which was also noticed in our previously published data [19].

Serine palmitoyltransferase 1 (SPTLC1), serine palmitoyltransferase 2 (SPTLC2), 3-ketodihydrosphingosine reductase (KDSR), ceramide synthase 2 (CerS2), ceramide synthase 4 (CerS4), ceramide synthase 6 (CerS6), dihydroceramide desaturase (DES), sphingosine kinase 1 (SPHK1), sphingosine kinase 2 (SPHK2), sphingosine-1-phosphate phosphatase (SPPase), acid ceramidase (ASAH1), neutral ceramidase (ASAH2), alkaline ceramidase (ASAH3), alkaline sphingomyelinase (Alk-SMase), neutral sphingomyelinase (N-SMase), phosphorylated insulin receptor substrate 1 (pIRS1(Ser636/Ser639)), phosphorylated phosphate and tensin homolog (pPTEN(Ser380)), phosphorylated protein kinase B (pAkt(Ser473)), phosphorylated glycogen synthase kinase 3α/β (pGSK3α/β(Ser21/Ser9)), phosphorylated Bcl-2-associated agonist of cell death (pBAD(Ser136)), phosphorylated mechanistic target of rapamycin (pmTOR(Ser2448), phosphorylated P70 ribosomal protein S6 kinase (pP70 S6 kinase(Thr389)), phosphorylated P90 ribosomal protein S6 kinase (pP90 S6 kinase), phosphorylated S6 ribosomal protein (pS6RP(Ser235/Ser236)) and glucose transporter 2 (GLUT2).

## 5. Conclusions

**Scheme 1:**
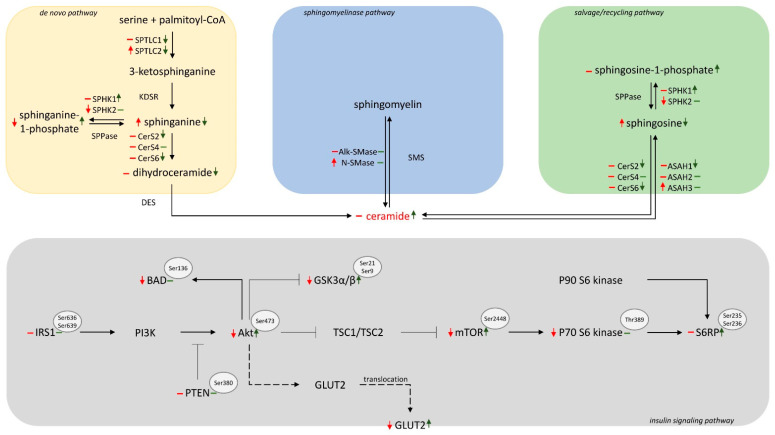
Graphical presentation of 8 week α-LA supplementation on the sphingolipid metabolism and insulin signaling pathways in the liver tissue of rats subjected to a high-fat diet. ↑—an increase, ↓—a decrease, –without change; red arrows represent high-fat feeding for eight weeks vs. control; green arrows represent the effects of 8-week α-LA administration vs. high-fat diet.

α-lipoic acid, as a naturally occurring antioxidant, may appear to have an effective therapeutic influence on the sphingolipid metabolism and insulin resistance during steatosis deterioration and progression into steatohepatitis. Our study presented that α-LA treatment provoked a decrease in sphinganine, dihydroceramide and sphingosine levels with an increase in the ceramide level. These alterations were correlated with the reduction in the hepatic-specific expression of SPTLC2, CerS2 and CerS6. Moreover, administering antioxidant to rats with MASLD induced by an HFD increased the phosphorylation of SFA and SFO, which resulted in increases in the content of SA1P and S1P, respectively. These results suggested new question about the role of this antioxidant in the sphingolipid balance under HFD feeding. In the further investigation, the use of a selective inhibitor of SPTLC, namely myriocin, and a selective inhibitor of CerS, namely fumonisin B1, can allow confirmation of which pathway, *de novo* or salvage, is mainly responsible for observed changes in the hepatic sphingolipid pool after α-lipoic acid administration. This study also demonstrated that the PI3K/Akt/mTOR signaling pathway is activated, particularly the phosphorylation of Akt and mTOR, after α-LA supplementation; thus, this antioxidant may alleviate glucose intolerance in the liver damage in HFD-subjected rats. Our results indicated a potential protective effect of α-lipoic acid on the disturbances in the sphingolipid metabolism and insulin transduction under HFD, which protects from steatosis deterioration (Scheme 1).

## Data Availability

Data is contained within the article.

## References

[1] Alharthi J., Eslam M. (2022). Biomarkers of Metabolic (Dysfunction)-Associated Fatty Liver Disease: An Update. J. Clin. Transl. Hepatol..

[2] Lee B.P., Dodge J.L., Terrault N.A. (2023). National Prevalence Estimates for Steatotic Liver Disease and Sub-Classifications Using Consensus Nomenclature. Hepatology.

[3] Johnston M.P., Patel J., Byrne C.D. (2020). Causes of Mortality in Non-Alcoholic Fatty Liver Disease (NAFLD) and Alcohol Related Fatty Liver Disease (AFLD). Curr. Pharm. Des..

[4] Gruben N., Shiri-Sverdlov R., Koonen D.P.Y., Hofker M.H. (2014). Nonalcoholic Fatty Liver Disease: A Main Driver of Insulin Resistance or a Dangerous Liaison?. Biochim. Biophys. Acta Mol. Basis Dis..

[5] Patterson R.E., Kalavalapalli S., Williams C.M., Nautiyal M., Mathew J.T., Martinez J., Reinhard M.K., McDougall D.J., Rocca J.R., Yost R.A. (2016). Lipotoxicity in Steatohepatitis Occurs despite an Increase in Tricarboxylic Acid Cycle Activity. Am. J. Physiol. Endocrinol. Metab..

[6] Gai Z., Gui T., Alecu I., Lone M.A., Hornemann T., Chen Q., Visentin M., Hiller C., Kullak-ublick S.H.G.A. (2020). Farnesoid X Receptor Activation Induces the Degradation of Hepatotoxic 1-Deoxysphingolipids in Non-Alcoholic Fatty Liver Disease. Liver Int..

[7] Zabielski P., Hady H.R., Chacinska M., Roszczyc K., Gorski J., Blachnio-Zabielska A.U. (2018). The Effect of High Fat Diet and Metformin Treatment on Liver Lipids Accumulation and Their Impact on Insulin Action. Sci. Rep..

[8] Chocian G., Chabowski A., Żendzian-Piotrowska M., Harasim E., Łukaszuk B., Górski J. (2010). High Fat Diet Induces Ceramide and Sphingomyelin Formation in Rat’s Liver Nuclei. Mol. Cell Biochem..

[9] Régnier M., Polizzi A., Guillou H., Loiseau N. (2019). Sphingolipid Metabolism in Non-Alcoholic Fatty Liver Diseases. Biochimie.

[10] Puri P., Baillie R.A., Wiest M.M., Mirshahi F., Choudhury J., Cheung O., Sargeant C., Contos M.J., Sanyal A.J. (2007). A Lipidomic Analysis of Nonalcoholic Fatty Liver Disease. Hepatology.

[11] Utzschneider K.M., Kahn S.E. (2006). The Role of Insulin Resistance in Nonalcoholic Fatty Liver Disease. J. Clin. Endocrinol. Metab..

[12] Park W.J., Song J.H., Kim G.T., Park T.S. (2020). Ceramide and Sphingosine 1-Phosphate in Liver Diseases. Mol. Cells.

[13] Svegliati-Baroni G., Pierantonelli I., Torquato P., Marinelli R., Ferreri C., Chatgilialoglu C., Bartolini D., Galli F. (2019). Lipidomic Biomarkers and Mechanisms of Lipotoxicity in Non-Alcoholic Fatty Liver Disease. Free Radic. Biol. Med..

[14] Pei K., Gui T., Kan D., Feng H., Jin Y., Yang Y., Zhang Q., Du Z., Gai Z., Wu J. (2020). An Overview of Lipid Metabolism and Nonalcoholic Fatty Liver Disease. Biomed. Res. Int..

[15] Gomes M.B., Negrato C.A. (2014). Alpha-Lipoic Acid as a Pleiotropic Compound with Potential Therapeutic Use in Diabetes and Other Chronic Diseases. Diabetol. Metab. Syndr..

[16] Smith A.R., Shenvi S.V., Widlansky M., Suh J.H., Hagen T.M. (2004). Lipoic Acid as a Potential Therapy for Chronic Diseases Associated with Oxidative Stress. Curr. Med. Chem..

[17] Moini H., Tirosh O., Park Y.C., Cho K.J., Packer L. (2002). R-α-Lipoic Acid Action on Cell Redox Status, the Insulin Receptor, and Glucose Uptake in 3T3-L1 Adipocytes. Arch. Biochem. Biophys..

[18] Hodun K., Sztolsztener K., Chabowski A. (2021). Antioxidants Supplementation Reduces Ceramide Synthesis Improving the Cardiac Insulin Transduction Pathway in a Rodent Model of Obesity. Nutrients.

[19] Zwierz M., Chabowski A., Sztolsztener K. (2023). α-Lipoic Acid—A Promising Agent for Attenuating Inflammation and Preventing Steatohepatitis in Rats Fed a High-Fat Diet. Arch. Biochem. Biophys..

[20] Lema A.G., Bekele B.M. (2023). Review on High Performance Liquid Chromatography Method of Development, Public Health Importance and Validation. Austin Chromatogr..

[21] Kim Y.R., Lee E.J., Shin K.O., Kim M.H., Pewzner-Jung Y., Lee Y.M., Park J.W., Futerman A.H., Park W.J. (2019). Hepatic Triglyceride Accumulation via Endoplasmic Reticulum Stress-Induced SREBP-1 Activation Is Regulated by Ceramide Synthases. Exp. Mol. Med..

[22] Kitatani K., Idkowiak-Baldys J., Hannun Y.A. (2008). The Sphingolipid Salvage Pathway in Ceramide Metabolism and Signaling. Cell Signal.

[23] Trayssac M., Hannun Y.A., Obeid L.M. (2018). Role of Sphingolipids in Senescence: Implication in Aging and Age-Related Diseases. J. Clin. Investig..

[24] Turpin S.M., Nicholls H.T., Willmes D.M., Mourier A., Brodesser S., Wunderlich C.M., Mauer J., Xu E., Hammerschmidt P., Brönneke H.S. (2014). Obesity-Induced CerS6-Dependent C16:0 Ceramide Production Promotes Weight Gain and Glucose Intolerance. Cell Metab..

[25] De La Monte S.M., Lyn-Cook L.E., Lawton M., Tong M., Silbermann E., Longato L., Jiao P., Mark P., Xu H., Wands J.R. (2011). Hepatic Ceramide May Mediate Brain Insulin Resistance and Neurodegeneration in Type 2 Diabetes and Non-Alcoholic Steatohepatitis. Adv. Alzheimer. Dis..

[26] Ishay Y., Nachman D., Khoury T., Ilan X.Y. (2020). The Role of the Sphingolipid Pathway in Liver Fibrosis: An Emerging New Potential Target for Novel Therapies. Am. J. Physiol. Cell Physiol..

[27] Prieto-Hontoria P.L., Pérez-Matute P., Fernández-Galilea M., Martínez J.A., Moreno-Aliaga M.J. (2011). Lipoic Acid Inhibits Leptin Secretion and Sp1 Activity in Adipocytes. Mol. Nutr. Food Res..

[28] Gault C.R., Obeid L.M., Hannun Y.A. (2010). An Overview of Sphingolipid Metabolism: From Synthesis to Breakdown. Adv. Exp. Med. Biol..

[29] Ma M.M., Chen J.L., Wang G.G., Wang H., Lu Y., Li J.F., Yi J., Yuan Y.J., Zhang Q.W., Mi J. (2007). Sphingosine Kinase 1 Participates in Insulin Signalling and Regulates Glucose Metabolism and Homeostasis in KK/Ay Diabetic Mice. Diabetologia.

[30] Li S., Kim H.E. (2021). Implications of Sphingolipids on Aging and Age-Related Diseases. Front. Aging.

[31] Newton J., Lima S., Maceyka M., Spiegel S. (2015). Revisiting the Sphingolipid Rheostat: Evolving Concepts in Cancer Therapy. Exp. Cell Res..

[32] Hajduch E., Lachkar F., Ferré P., Foufelle F. (2021). Clinical Medicine Roles of Ceramides in Non-Alcoholic Fatty Liver Disease. J. Clin. Med..

[33] Park J.-W., Park W.-J., Kuperman Y., Boura-Halfon S., Pewzner-Jung Y., Futerman A.H. (2012). Ablation of Very Long Acyl Chain Sphingolipids Causes Hepatic Insulin Resistance in Mice Due to Altered Detergent-Resistant Membranes. Hepatology.

[34] Wang X., Ye T., Li M., Li X., Qiang O., Tang C., Liu R. (2017). Effects of Octreotide on Hepatic Glycogenesis in Rats with High Fat Diet-induced Obesity. Mol. Med. Rep..

[35] Heydemann A., González-Vega M., Berhanu T.K., Mull A.J., Sharma R., Holley-Cuthrell J. (2016). Hepatic Adaptations to a High Fat Diet in the MRL Mouse Strain Are Associated with an Inefficient Oxidative Phosphorylation System. Jacobs J. Diabetes Endocrinol..

[36] Ma Z., Chu L., Liu H., Wang W., Li J., Yao W., Yi J., Gao Y. (2017). Beneficial Effects of Paeoniflorin on Non-Alcoholic Fatty Liver Disease Induced by High-Fat Diet in Rats. Sci. Rep..

[37] Sztolsztener K., Konstantynowicz-Nowicka K., Harasim-Symbor E., Chabowski A. (2021). Time-Dependent Changes in Hepatic Sphingolipid Accumulation and PI3K/Akt/MTOR Signaling Pathway in a Rat Model of NAFLD. Int. J. Mol. Sci..

[38] Tardif J.-C., Rhéaume E. (2008). Lipoic Acid Supplementation and Endothelial Function. Br. J. Pharmacol..

[39] Smith A.R., Visioli F., Frei B., Hagen T.M. (2008). Lipoic Acid Significantly Restores, in Rats, the Age-Related Decline in Vasomotion. Br. J. Pharmacol..

[40] Gupte A.A., Bomhoff G.L., Morris J.K., Gorres B.K., Geiger P.C. (2009). Lipoic Acid Increases Heat Shock Protein Expression and Inhibits Stress Kinase Activation to Improve Insulin Signaling in Skeletal Muscle from High-Fat-Fed Rats. J. Appl. Physiol..

[41] Cho C.S., Kowalsky A.H., Lee J.H. (2020). Pathological Consequences of Hepatic MTORC1 Dysregulation. Genes.

[42] Kimball S.R., Ravi S., Gordon B.S., Dennis M.D., Jefferson L.S. (2015). Amino Acid–Induced Activation of MTORC1 in Rat Liver Is Attenuated by Short-Term Consumption of a High-Fat Diet. J. Nutr..

[43] Peng P., Zhang X., Qi T., Cheng H., Kong Q., Liu L., Cao X., Ding Z. (2020). Alpha-lipoic Acid Inhibits Lung Cancer Growth via MTOR-mediated Autophagy Inhibition. FEBS Open Bio..

[44] Ruvinsky I., Meyuhas O. (2006). Ribosomal Protein S6 Phosphorylation: From Protein Synthesis to Cell Size. Trends Biochem. Sci..

[45] Emma M.R., Augello G., Cusimano A., Azzolina A., Montalto G., McCubrey J.A., Cervello M. (2020). GSK-3 in Liver Diseases: Friend or Foe?. Biochim. Et Biophys. Acta (BBA)—Mol. Cell Res..

